# Relapsing Polychondritis in a Patient With Auricular Chondritis and Inflammatory Bowel Disease: A Case Report With Literature Review

**DOI:** 10.7759/cureus.31738

**Published:** 2022-11-21

**Authors:** David D Bickford, Thomas Ritter, Pinky Jha, Hari R Paudel

**Affiliations:** 1 Internal Medicine, Medical College of Wisconsin, Wauwatosa, USA; 2 Family Medicine, Medical College of Wisconsin, Milwaukee, USA

**Keywords:** extraintestinal manifestation, relapsing polychondritis, ulcerative colitis, crohn's disease, inflammatory bowel disease, auricular chondritis

## Abstract

Relapsing polychondritis (RP) most commonly presents as inflammation and degeneration of cartilaginous tissue in the auricles, nasal septum, and lungs (in severe instances). RP is a rare autoimmune condition associated with other autoimmune diseases in 30% of cases. The prevalence of gastrointestinal involvement with RP is tenuous; however, there is a growing collection of case studies associating auricular chondritis with concomitant inflammatory bowel disease (IBD), including both ulcerative colitis and Crohn’s disease. We report the case of a 35-year-old patient presenting with autoimmune pancreatitis, with a past medical history of Crohn’s disease, primary sclerosing cholangitis (PSC), and suspected RP. Although RP is rare, the disease’s multiple clinical presentations and recurrent episodic nature can cause significant diagnostic delays and are often overlooked by physicians. Thus, low disease prevalence may be due to under-recognition and under-reporting of disease symptoms. As RP is a clinical diagnosis, increased awareness of the disease presentation and clinical characteristics may increase disease recognition and improve treatment outcomes.

## Introduction

Relapsing polychondritis (RP) is an autoimmune disease associated with the immune-mediated destruction of cartilaginous structures. Less common manifestations include ocular complications (anterior scleritis, episcleritis, conjunctivitis), nasal chondritis, polyarthralgia, and dermatologic manifestations such as aphthous ulcers, erythema nodosum, and distal skin ulceration (thought to be attributed to associated local vasculitis). The rate of RP is rare, with about 3.5 cases per million per year in the United States [1]. Over 1000 cases of RP have been published globally with a growing subset of cases including patients with co-existing Crohn’s disease (12 patients) and ulcerative colitis (18 patients); current case report not included [2].

More severe inflammatory bowel disease (IBD) is often accompanied by extraintestinal manifestations (EIM), with a prevalence range of 25-40% [3]. EIMs are capable of affecting any system in the body and can be associated with active colitis (including arthralgias, dermatologic, and ocular manifestations) or present with or without flare (such as osteoporosis or hepatobiliary disease) [4]. Notably, arthralgias, ocular inflammation, aphthous ulcers, and erythema nodosum can present in both RP and IBD, suggesting a similar autoimmune etiology [1]. Furthermore, the presence of one EIM has been shown to increase the likelihood of presentation for additional manifestations and close monitoring of new symptoms should be maintained for early recognition to guide therapy.

Due to the complex autoimmune manifestations of both IBD and RP, initial presenting symptoms might be primary or secondary to either condition. Auricular chondritis may precede the onset of gastrointestinal symptoms and present as an initial diagnostic clue for IBD patients [5]. Since 30% of RP cases are associated with other autoimmune diseases, and a growing number of cases are associated with IBD, it is difficult to determine whether IBD patients have concomitant RP or if chondritis is an additional EIM. Thus, further understanding of the role chondritis plays in RP and IBD is needed for early disease detection and decreased mortality.

## Case presentation

Our patient was a 35-year-old Caucasian male with a history of Crohn’s disease diagnosed in 2002, primary sclerosing cholangitis (PSC), and recurrent pancreatitis admitted for abdominal pain. He reported cramp-like, post-prandial peri-umbilical, and bilateral lower abdominal pain for the last 7-8 days, which had been progressively worsening. His pain worsened with eating and activity and typically radiated to his lower back. The patient denied fever, chills, jaundice, rectal bleeding, melena, fecal urgency, pruritis, nausea, vomiting, or diarrhea. He reported no alcohol abuse, gallstone symptoms, or history of hypertriglyceridemia. The patient was afebrile, intermittently bradycardic, and normotensive. The basic metabolic panel (BMP) and complete blood count (CBC) were unremarkable. Additional pertinent labs can be found in Table 1.

**Table 1:** Pertinent lab values including elevated liver enzymes, amylase, lipase, and inflammatory markers.

Lab Test	Result	Reference Range
Alkaline phosphatase	863 U/L	40 - 129 U/L
Alanine aminotransferase (ALT)	284 U/L	8 - 66 U/L
Aspartate aminotransferase (AST)	176 U/L	13 - 44 U/L
Albumin	3.9 g/dL	3.8 - 5.0 g/dL
Total protein	8.5 g/dL	6.1 - 8.2 g/dL
Total bilirubin	1.0 mg/dL	0.2 - 1.2 mg/dL
Direct bilirubin	0.4 mg/dL	≤ 0.3 mg/dL
Gamma-glutamyl transferase (GGT)	533 U/L	10 - 71 U/L
Lipase	256 U/L	13 - 60 U/L
Amylase	228 U/L	28 - 100 U/L
Erythrocyte sedimentation rate (ESR)	47 mm/hr	0 - 15 mm/hr
C-reactive protein (CRP)	2.89 mg/dL	< 0.5 mg/dL
Immunoglobulin G-4 (IgG4)	32 mg/dL	1 - 123 mg/dL
Immunoglobulin G (IgG)	1447 mg/dL	700 - 1600 mg/dL
Smooth muscle antibody IgG	1:160	< 1:20

**Figure 1:**
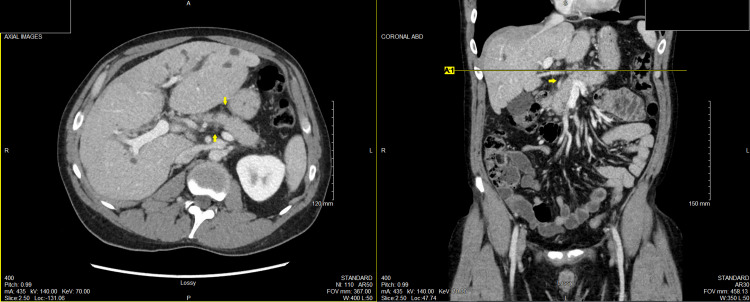
CT A/P with acute interstitial pancreatitis, fat stranding, and edema around the pancreatic parenchyma.

The patient was COVID-19 negative. Hepatitis B surface antigen, hepatitis B core antibody IgM, and hepatitis C antibody were all nonreactive. CT A/P showed suspicion of acute interstitial pancreatitis with generalized mild inflammatory fat stranding and edema within the pancreatic parenchyma (Figure 1). The patient was diagnosed with acute interstitial pancreatitis, likely autoimmune and secondary to PSC.

Family and social history were non-contributory. On physical exam, his ear appeared misshapen, and he reported occasional erythema, pain, and red color of the outer ears and occasional nasal cartilage pain since childhood that would resolve spontaneously. These episodes lasted for several days to a week before resolving. The hearing was intact and normal. The patient also noted frequent aphthous ulcers as a child and these symptoms occurred prior to his onset of IBD. No auricular chondritis relapses occurred after initiating prednisone bursts and infliximab treatment for his Crohn’s disease. Since the patient did not present in an acute auricular or nasal chondritis flare, biopsies were not collected. Due to his relapsing history, multiple sites of involvement, and positive response to corticosteroids and infliximab, a clinical diagnosis of auricular chondritis secondary to RP was suggestive. The patient was advised to monitor for additional flares and schedule an outpatient appointment with rheumatology as needed.

## Discussion

Auricular chondritis is the most common presentation of RP, with nasal chondritis a close second, and leads to acute, erythematous, tender inflammation of the external ear with sparing of the lobule. Since the auricular cartilage has no direct blood supply, it receives all nutrients via diffusion from the perichondrium; thus, repeat episodes of inflammation and swelling may damage the outer ear cartilage. Subsequent fibrocartilage overgrowth can lead to permanent deformity known as cauliflower ear. Additionally, auricular chondritis is associated with extensive calcification, limp pinna (i.e., forward listening ear), and highly visible underlying vasculature due to thinning of the cartilage (i.e., blue ear sign) [6]. The damage of auricular chondritis can be progressive with relapse and lead to less common manifestations, including temporary conductive hearing loss and tinnitus. However, it is noted that hearing loss is not due to the disease process itself but instead caused by inflammation of the auditory meatus [7]. Permanent hearing loss is possible with severe disease presentations and is thought to be a consequence of vasculitis in the vestibular or cochlear branch of the internal auditory artery [8].

RP is caused by damaged cartilage, which may release collagen fragments leading to circulating immune complex formation. The disease process is thought to be autoimmune, with circulating antibodies to type II collagen found in 30-60% of cases [6], as well as immunoglobulin G (IgG), immunoglobulin A (IgA), and complement component 3 (C3) seen in affected cartilage by direct immunofluorescence examination [5]. However, specific lab values or histological markers are not currently available to make a final diagnosis of RP. Additionally, erythrocyte sedimentation rate (ESR) and C-reactive protein (CRP) may be helpful for the initial clinical evaluation of patients with suspected RP as a surrogate for disease activity and treatment response. Acute episodes last from a few days to several weeks. Numerous studies have also shown some value in the use of PET imaging for early disease recognition and tissue extension [2], however to date; diagnosis is primarily made clinically using one of three accepted clinical criteria established by McAdam et al. (1976) [9] and revised by Damiani & Levine (1979) [10] and Michet et al. (1986) [11]. The most recent criteria suggested by Michet et al. recommend two of three primary symptoms, including auricular cartilage inflammation, nasal cartilage inflammation, or laryngotracheal inflammation for diagnosis. Patients may also meet diagnostic criteria by having one of three primary symptoms and two secondary symptoms, including ocular inflammation, hearing loss, vestibular dysfunction, or seronegative arthritis [11]. 

With the growing number of case reports and the pathophysiology of IBD and RP involving cellular and humoral immunity, it’s tempting to consider auricular chondritis as a potential extraintestinal manifestation of IBD. However, based on our current understanding of the mechanisms behind these diseases, we cannot make a robust determination. For example, RP is due to a Th1 phenotype autoimmune response to type II collagen, causing a pro-inflammatory, IL-8, and TNF-alpha-driven disease cascade [7], [12]. Crohn’s disease is a Th1-like condition, often considered to be driven by increased production of IFN-y, while Ulcerative Colitis is considered an atypical Th2 response secreting IL-5, IL-6, and IL-13 [13]. Additionally, RP and IBD tend to interact with different types of collagens present in the ear/nose versus the gastrointestinal tract, with collagen II, IX, and XI being primarily implicated in RP, and collagen I, III, and V found in IBD. Collagen V has been shown to be associated with intestinal strictures [14]. TNF-alpha has also been shown to be universally increased in all three diseases, including both Crohn's disease and Ulcerative Colitis [15]. Although the pathogenesis is likely different, RP and the extraintestinal manifestations of IBD tend to both improve with anti-TNF therapy (Infliximab) [16], [17]. Thus, TNF-alpha may have a fundamental role in the pathogenesis of RP and the extraintestinal manifestations of IBD [4].

Both IBD and RP have progressive disease severity with recurrence and the goal of treatment is to resolve symptoms, preserve the cartilaginous integrity, and maintain long-term inhibition of immunity to prevent future recurrence. Unfortunately, clinical research on definitive treatments for relapsing polychondritis is scarce, and treatment plans are primarily derived from case-based evidence. Current empiric treatment is determined by disease severity and includes NSAIDs, colchicine, dapsone, and low-dose corticosteroids for mild forms of RP. For severe cases, first-line treatment includes oral prednisone or IV pulse methylprednisone. With steroids, the erythema of the preauricular and postauricular soft tissue should recede dramatically within 24 hours. Azathioprine or methotrexate can also be used for second-line treatment. In severe cases involving organ-threatening complications of RP, TNF inhibitors like Infliximab should be considered. Further, a recent meta-analysis with Infliximab in RP [17] reported success in treating co-existing RP in patients with Crohn’s disease [18]. Therefore, Infliximab may be considered a second-line therapy in conjunction with methotrexate. Switching to another biologic (tocilizumab or abatacept) should be considered in cases where Infliximab has lost efficacy. Further research is needed to determine standardized treatment guidelines for auricular relapsing polychondritis, and its relationship to IBD.

**Table 2:** Case series review for IBD, PBC and PSC, and relapsing polychondritis. IBD: inflammatory bowel disease; PSC: primary sclerosing cholangitis; PBC: primary biliary cirrhosis

Co-existing Disease	Primary Author and Date [reference], (# cases)	Total # Cases
Ulcerative colitis	Rosen et al. 1969 [19], (1); McAdam et al. 1976 [9], (1); Prévot et al. 1981 [20], (1); Asuncion et al. 1994 [21], (1); Zeuner et al. 1997 [22] (2); Wasiak et al. 1996 [23], (1); Benito Calavia et al. 1997 [24], (1); Demerjian et al. 2001 [25], (1); Kawano et al. 2001 [26], (1); Andre et al. 2007 [27], (1); Almadi et al. 2010 [28], (1); Karmacharya et al. 2014 [29], (1); Mydlak et al. 2017 [30], (1); Tajiri et al. 2019 [31], (1); Hanioka et al. 2021 [13], (1); Hutto et al. 2021 [32], (2)	18
Crohn’s disease	Haigh et al. 1987 [33], (1); Pazirandeh et al. 1988 [34], (1); Zeuner et al. 1997 [22], (1); Touma et al. 1996 [35], (1); Piette et al. 1999 [36], (2); Francès et al. 2001 [37], (2); Vicente et al. 2008 [38](1); Naidu et al. 2018 [39], (1); Jung et al. 2021 [18], (1); Grygiel-Górniak 2021 [40], (1)	12
PBC	Conn et al. 1982 [41], (1)	1
PSC	Mydlak et al. 2017 [30], (1)	1

## Conclusions

RP is a complex autoimmune disease with multisystem complications, including a possible association with IBD. This case report and literature review identified 30 cases of RP with concomitant IBD, and to our knowledge, this is the 31st case to add to the limited literature. Since criteria for RP diagnosis are based on clinical presentation, recognition of common disease characteristics is critical to increasing RP awareness and improving treatment outcomes. Additionally, with a growing number of cases reported, further research is needed to determine the autoimmune etiology of RP to better understand its relationship with IBD.
